# Neuroprotective effects of amiodarone in a mouse model of ischemic stroke

**DOI:** 10.1186/s12871-017-0459-3

**Published:** 2017-12-08

**Authors:** Masakazu Kotoda, Tadahiko Ishiyama, Kazuha Mitsui, Sohei Hishiyama, Takashi Matsukawa

**Affiliations:** 1 Department of Anesthesiology, Faculty of Medicine, University of Yamanashi, 1110 Shimokato, Chuo, Yamanashi 409-3898 Japan; 2 Surgical Center, University of Yamanashi Hospital, University of Yamanashi, 1110 Shimokato, Chuo, Yamanashi 409-3898 Japan

**Keywords:** Amiodarone, Pre-treatment, Stroke, Neuroprotection

## Abstract

**Background:**

Ion channels play a crucial role in the development of ischemic brain injury. Recent studies have reported that the blockade of various types of ion channels improves outcomes in experimental stroke models. Amiodarone, one of the most effective drugs for life-threatening arrhythmia, works as a multiple channel blocker and its characteristics cover all four Vaughan-Williams classes. Although it is known that amiodarone indirectly contributes to preventing ischemic stroke by maintaining sinus rhythm in patients with atrial fibrillation, the direct neuroprotective effect of amiodarone has not been clarified. The purpose of this study was to investigate the direct effect of amiodarone on ischemic stroke in mice.

**Methods:**

Focal cerebral ischemia was induced via distal permanent middle cerebral artery occlusion (MCAO) in adult male mice. The amiodarone pre-treatment group received 50 mg/kg of amiodarone 1 h before MCAO; the amiodarone post-treatment groups received 50 mg/kg of amiodarone immediately after MCAO; the control group received vehicle only. In addition, the sodium channel opener veratrine and selective beta-adrenergic agonist isoprotelenol were used to elucidate the targeted pathway. Heart rate and blood pressure were monitored perioperatively. Infarct volume analysis was conducted 48 h after MCAO. The body asymmetry test and the corner test were used for neurological evaluation.

**Results:**

Amiodarone pre-treatment and post-treatment reduced the heart rate but did not affect the blood pressure. No mice showed arrhythmia. Compared with the control group, the amiodarone pre-treatment group had smaller infarct volumes (8.9 ± 2.1% hemisphere [mean ± SD] vs. 11.2 ± 1.4%; *P* < 0.05) and improved functional outcomes: lower asymmetric body swing rates (52 ± 17% vs. 65 ± 18%; *P* < 0.05) and fewer left turns (7.1 ± 1.2 vs. 8.3 ± 1.2; *P* < 0.05). In contrast, amiodarone post-treatment did not improve the outcomes after MCAO. The neuroprotective effect of amiodarone pre-treatment was abolished by co-administration of veratrine but not by isoproterenol.

**Conclusions:**

Amiodarone pre-treatment attenuated ischemic brain injury and improved functional outcomes without affecting heart rhythm and blood pressure. The present results showed that amiodarone pre-treatment has neuroprotective effects, at least in part, via blocking the sodium channels.

## Background

Ion channels play a crucial role in the development of ischemic brain injury [1]. After ischemic insult, Na^+^-K^+^- adenylpyrophosphatase (Na^+^-K^+^-ATPase) becomes inactivated due to the depletion of cellular ATP. Intracellular Na^+^ accumulates and the brain cell membrane becomes depolarized. Excessive glutamate release and subsequent activation of voltage-gated Na^+^ and Ca^2+^ channels occur, resulting in neuronal hyperexcitability with further accumulation of intracellular Na^+^ and Ca^2+^. As a result, cellular necrosis due to osmotic swelling occurs [1]. Recently, many studies have reported that the blockade of the Na^+^ and Ca^2+^ channels improves outcomes in experimental models of ischemic stroke [1–5].

Amiodarone, one of the most effective drugs for the treatment of life-threatening arrhythmia, works as a multiple ion channel blocker and its characteristics cover all four Vaughan-Williams classes [6]. Besides its inhibitory effects on K^+^ channels, amiodarone also has inhibitory effects on the Na^+^ and Ca^2+^ channels [6]. Considering amiodarone’s pharmacological profile as a multiple ion channel blocker, there is a possibility that amiodarone has neuroprotective effects on the ischemic brain. It is known that amiodarone indirectly contributes to the prevention of ischemic stroke by maintaining sinus rhythm in patients with atrial fibrillation [7]. However, the direct neuroprotective effect of amiodarone has not been clarified.

With the above in mind, the purpose of this study was to evaluate the neuroprotective effect of amiodarone on ischemic brain injury. We tested whether pre-treatment or post-treatment with amiodarone effectively attenuates ischemic brain injury because it can be used both before and after ischemic insult caused by life-threatening or refractory arrhythmia in clinical situations. Our hypothesis was that pre-treatment and post-treatment with amiodarone attenuate ischemic brain injury. We examined the following outcomes with amiodarone treatment: infarct volume, neurological function, hemodynamics, and arrhythmias.

## Methods

Experiments were conducted in accordance with the National Institutes of Health (NIH) guidelines for the care and use of laboratory animals. The experimental protocol was reviewed and approved by the University of Yamanashi Animal Care Committee.

### Animals

Male C57B6 mice (age, 8–12 weeks old; weight, 20–25 g) were purchased from Japan SLC (Tokyo, Japan). The mice were housed at 23 ± 2 °C under a 12-h light–dark cycle with free access to standard food and water.

### Focal cerebral ischemia

Focal cerebral ischemia was induced via distal permanent middle cerebral artery occlusion (MCAO) as previously described [8]. Briefly, animals were anesthetized with 2% isoflurane in a 30% O_2_/68% N_2_ mixture. Mice were placed in the lateral positon and a skin incision was made between the left orbit and ear. A 2-mm burr hole was made using a dental drill (Ideal Microdrill; Bio Research, Nagoya, Japan) over the left middle cerebral artery (MCA). The distal portion of the left MCA was exposed and coagulated using a small vessel cauterizer (Fine Science Tools, Inc., Foster City, CA), followed by transection of the artery. A laser Doppler flowmeter (FLO-C1; Omegaflo, Tokyo, Japan) was used to monitor the cerebral blood flow in the brain area supplied by the left MCA. Mice that showed less than an 85% decrease in cerebral blood flow were excluded. Rectal temperature was monitored and maintained at 37 ± 0.5 °C using a heating pad until recovery from anesthesia. Mice were euthanized 48 h after distal permanent MCAO, followed by infarct volume analysis.

### Treatment

Amiodarone (Sanofi K.K., Tokyo, Japan), veratrine (Santa Cruz Biotecnology, Dallas, USA), and isoproterenol (Kowa Pharmaceutical, Tokyo, Japan) were dissolved or suspended in normal saline. All drugs were freshly prepared before use and administered intraperitoneally.

The amiodarone pre-treatment group (*n* = 15) received a single-bolus injection of 50 mg/kg amiodarone suspended in 0.5 ml of saline 1 h prior to MCAO and 0.5 ml of saline immediately after MCAO. The amiodarone post-treatment group (*n* = 15) received 0.5 ml of saline 1 h prior to MCAO and the same dosage of amiodarone immediately after MCAO. The amiodarone + veratrine group (*n* = 6) received 50 mg/kg amiodarone and 0.125 mg/kg veratrine dissolved in 0.5 ml of saline 1 h prior to MCAO and 0.5 ml of saline immediately after MCAO. The amiodarone + isoproterenol group (*n* = 6) received 50 mg/kg amiodarone and 0.04 mg/kg isoproterenol dissolved in 0.5 ml of saline 1 h prior to MCAO and 0.5 ml of saline immediately after MCAO. The control group (*n* = 15) received 0.5 ml of normal saline 1 h prior to MCAO and immediately after MCAO.

### Hemodynamic measurements

Heart rate, heart rhythm, and non-invasive blood pressure were measured 1 h before MCAO (baseline). These parameters were monitored for 1 h before and after MCAO and measured 48 h after MCAO.

### Measurement of infarct volume

Forty-eight hours after MCAO and after hemodynamic measurements, mice were deeply anesthetized with 5% isoflurane and transcardially perfused with 1% phosphate-buffered saline. Brains were removed and coronal slices with a thickness of 2 mm were prepared. Brain slices were immersed in 2% 2,3,5-Triphenyltetrazolium chloride (TTC; Sigma Aldrich, St. Louis, MO) solution, and incubated at 37 °C for 15 min. The area of infarction was traced and measured using image analysis software (ImageJ; National Institutes of Health, Bethesda, MD). The infarct area was calculated as follows to correct for edema: [1 – (total ipsilateral hemisphere – infarct region) / total contralateral hemisphere] × 100% [9]. Total infarct volume was calculated as the sum of all infarct areas multiplied by section thickness.

### Neurological evaluations

Neurological function and deterioration were assessed by a blinded observer using the body asymmetry test [10] and the corner test [11] as previously described. The behavioral tests were conducted 48 h after MCAO, followed by infarct volume analysis.

During the body asymmetry test, animals were suspended by their tails and the direction of head movement was recorded [10]. The trials were repeated 20 times. Between each trial, animals were allowed to move freely in the cage for 30 s. Lateral head movements, on either the contralateral side or the ipsilateral side, were counted and normalized as follows: (head movements toward the contralateral side – 10) / 10 × 100%. The mice with damage to the cortex are expected to show head movements toward the contralateral side. This test was performed to assess motor asymmetry [12].

During the corner test, a corner was made by attaching two boards (30 cm × 20 cm × 1 cm) at an angle of 30°. A small opening was made along the joint to encourage entry into the corner. Animals were placed midway from the corner. When the animals reached the corner and their vibrissae were stimulated, they reared upward and then turned to either side. The number of left (ipsilateral side) turns during 10 trials was recorded. The mice with damage to the left cortex are expected to turn more toward the left (ipsilateral) side. This test was used to assess the sensorimotor and postural function [11, 12].

### Statistical analysis

All values are presented as means ± standard deviation (SD). Subgroup comparisons were analyzed using one-way analysis of variance (ANOVA), followed by the Dunnett test. Hemodynamic data were compared among groups by two-way ANOVA for repeated measures. Statistical analysis was performed using Prism 6 software (GraphPad Software, San Diego, CA). *P* < 0.05 was considered statistically significant.

The sample size of 15 mice per group was sufficient to provide 80% power with an α level of 0.05 to detect a mean difference of 2% in infarct volume.

## Results

### Hemodynamic measurements

As shown in Table 1, the amiodarone pre-treatment group, amiodarone post-treatment group, and amiodarone and veratrine group showed lower heart rates compared to the control group [Table 1]. Compared with the control group, the amiodarone pre-treatment group showed an 18.7% decrease in heart rate at the time of MCAO, a 15.6% decrease at 1 h after MCAO, and an 8.2% decrease at 48 h after MCAO. The post-treatment group showed a 20.9% decrease in heart rate at 1 h after MCAO and a 6.9% decrease at 48 h after MCAO. The amiodarone + veratrine group showed a 9.8% decrease in heart rate at the time of MCAO and a 19.2% decrease at 1 h after MCAO. The amiodarone + isoproterenol group showed a 16.3% decrease in heart rate at 1 h after MCAO (*P* < 0.05 for all data). No animal demonstrated arrhythmia. There was a trend toward slightly lower systolic blood pressure and diastolic blood pressure in the pre-treatment and the post-treatment groups. However, the difference was not statistically significant (*P* = 0.066 at 0 h; *P* = 0.051 at 1 h; and *P* = 0.595 at 48 h) [Table 1].

**Table 1:** Hemodynamic measurements

Group		SBP	DBP	HR
		(mmHg)	(mmHg)	(beats/min)
Control	-1 h (Baseline)	109 ± 6	64 ± 6	469 ± 10
	0 h (MCAO)	106 ± 7	63 ± 6	470 ± 14
	1 h	124 ± 5	62 ± 8	526 ± 24
	48 h	116 ± 7	63 ± 11	490 ± 15
Amiodarone Pre-treatment	-1 h (Baseline)	107 ± 8	64 ± 5	477 ± 11
	0 h (MCAO)	102 ± 9	60 ± 7	382 ± 23*
	1 h	119 ± 8	60 ± 8	444 ± 28*
	48 h	113 ± 6	64 ± 7	450 ± 22*
Amiodarone Post-treatment	-1 h (Baseline)	108 ± 6	63 ± 5	474 ± 17
	0 h (MCAO)	109 ± 7	63 ± 6	471 ± 13
	1 h	117 ± 11	59 ± 12	416 ± 12*
	48 h	116 ± 13	64 ± 8	456 ± 18*
Amiodarone Pre-treatment + Veratrine	-1 h (Baseline)	110 ± 9	63 ± 7	485 ± 24
	0 h (MCAO)	114 ± 11	64 ± 8	424 ± 14*
	1 h	119 ± 9	63 ± 7	425 ± 16*
	48 h	113 ± 7	68 ± 4	491 ± 23
Amiodarone Pre-treatment + Isoproterenol	-1 h (Baseline)	109 ± 8	64 ± 9	465 ± 20
	0 h (MCAO)	100 ± 6	58 ± 4	472 ± 16
	1 h	110 ± 6	65 ± 4	440 ± 31*
	48 h	106 ± 8	64 ± 4	471 ± 37

Heart rate was lower in the amiodarone pre-treatment group, amiodarone post-treatment group, and amiodarone + veratrine group than in the control mice at the time of MCAO

There was a trend toward slightly lower systolic blood pressure and diastolic blood pressure for animals that received 50 mg/kg amiodarone; however, the difference was not statistically significant (*P < 0.05 vs. control)

*Abbreviations*: *SBP* systolic blood pressure, *DBP* diastolic blood pressure, *HR* heart rate, *MCAO* middle cerebral artery occlusion

### Infarct volume analysis

**Fig. 1:**
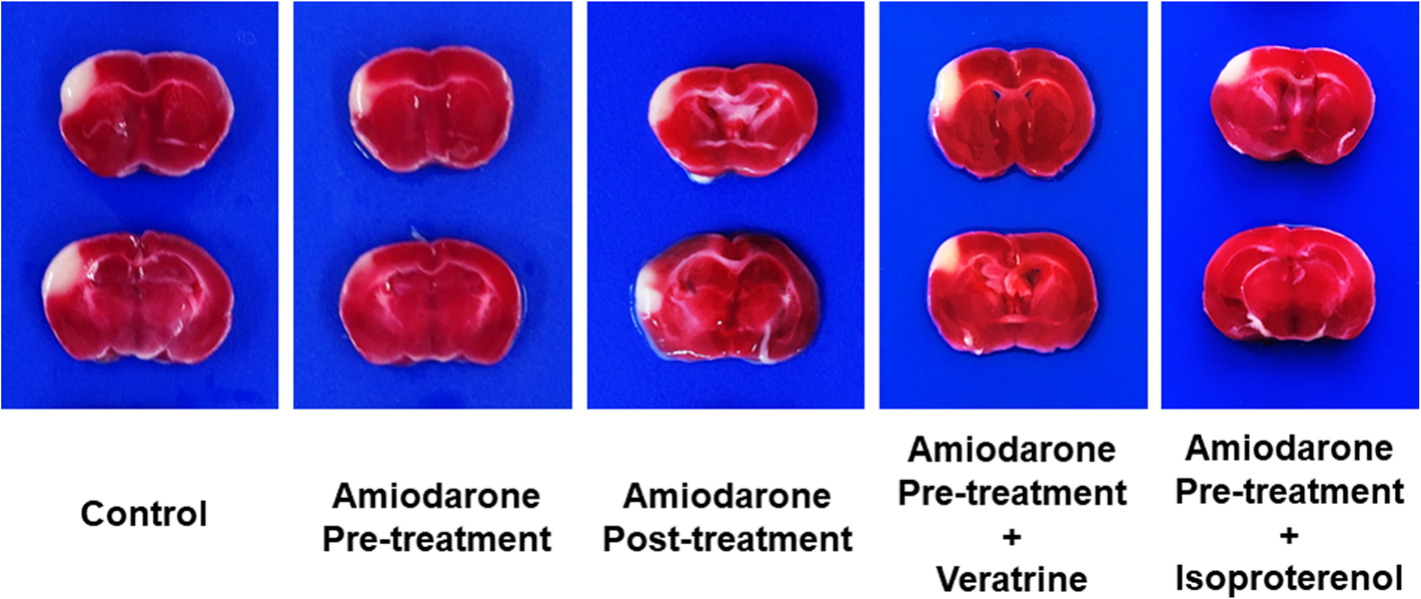
TTC staining. Representative 2,3,5-Triphenyltetrazolium chloride (TTC)-stained corresponding coronal brain sections are shown. The amiodarone pre-treatment group and amiodarone pre-treatment + isoproterenol group, but not the amiodarone post-treatment group and amiodarone pre-treatment + veratrine group, had smaller infarct areas

**Fig. 2:**
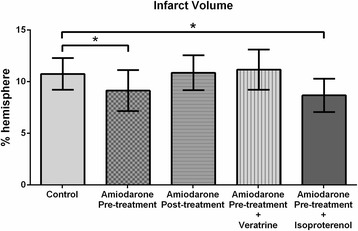
Infarct volume analysis. Reduced infarct volumes were observed in the amiodarone pre-treatment group and amiodarone pre-treatment + isoproterenol group but not in the post-treatment group and amiodarone pre-treatment + veratrine group (*n* = 6–15 / group). *P < 0.05 vs. control

Representative TTC-stained coronal brain sections are shown in Fig. 1. Compared with the control group, the amiodarone pre-treatment group had smaller infarct volume (8.9 ± 2.1% hemisphere [mean ± SD] vs. 11.2 ± 1.4%; *P* < 0.05; 95% confidence interval [CI]: 0.15–3.1). In contrast, amiodarone post-treatment did not reduce infarct volume (10.9 ± 1.8% vs. 11.2 ± 1.4%; 95% CI: -1.6–1.3) [Fig. 2]. The neuroprotective effect of amiodarone pre-treatment was abolished by co-administration of veratrine (11.6 ± 0.9% vs. 11.2 ± 1.4%; 95% CI: -2.3–1.5) Isoproterenol co-administration did not inhibit the neuroprotective effect of amiodarone pre-treatment (8.7 ± 1.9% vs. 11.2 ± 1.4%; *P* < 0.05; 95% confidence interval [CI]: 0.1–4.0).

### Behavioral tests

**Fig. 3:**
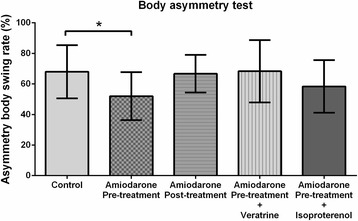
The body asymmetry test. The amiodarone pre-treatment group showed lower asymmetric body swing rates (*n* = 6–15 / group). *P < 0.05 vs. control

**Fig. 4:**
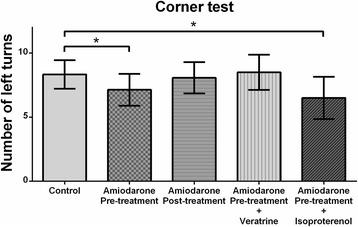
The corner test. Fewer left turns were observed in the amiodarone pre-treatment group and amiodarone pre-treatment + isoproterenol group (n 6–15 / group). *P < 0.05 vs. control

Amiodarone pre-treatment (but not post-treatment) improved functional outcomes of the behavioral tests [Figs. 3 and 4]. During the body asymmetry test, the amiodarone pre-treatment group had lower asymmetric body swing rates (52 ± 17% vs. 65 ± 18%; *P* < 0.05; 95% CI: 1–31) [Fig. 3]. During the corner test, fewer left turns were observed in the amiodarone pre-treatment group (7.1 ± 1.2 vs. 8.3 ± 1.2; *P* < 0.05; 95% CI: 0.0–2.4) [Fig. 4]. Amiodarone post-treatment did not cause any improvement in the results of these tests (67 ± 13% vs. 65 ± 18 and 95% CI: -13–16 for the body asymmetry test; 8.1 ± 1.3 vs. 8.3 ± 1.2 and 95% CI: -0.9–1.4 for the corner test). The functional improvement observed in the amiodarone pre-treatment group during the behavioral tests was abolished by co-administration of veratrine. (68 ± 20% vs. 65 ± 18 and 95% CI: -19–19 for the body asymmetry test; 8.5 ± 1.4 vs. 8.3 ± 1.2 and 95% CI: -1.7–1.4 for the corner test). The amiodarone + isoproterenol group had fewer left turns during the corner test compared with the control group (6.5 ± 1.6 vs. 8.3 ± 1.2; *P* < 0.05; 95% CI: 0.3–3.4) [Fig. 4], but did not show significant difference during the body asymmetry test (58 ± 17% vs. 65 ± 18%; 95% CI: -10–29) [Fig. 3].

## Discussion

In the present study, we found that amiodarone administered before ischemic brain insult lessened the infarct volume and improved neurological function.

Systemically administered amiodarone passes the blood–brain barrier [13, 14] and can exert its pharmacological effects on the central nervous system [15]. The pharmacological profile of amiodarone is complex. It works as a multiple ion channel blocker and has inhibitory effects on Na^+^, Ca^2+^, and K^+^ channels, Na^+^-K^+^-ATPase, and Na^+^/Ca^2+^ exchanger. [6, 16, 17] Previous studies have suggested the neuroprotective effect of various types of ion channel blockers including Na^+^ [2, 5], Ca^2+^ [18], and K^+^ blockers [3, 4]. Blockade of Na^+^ or Ca^2+^ channels prevents hyperexcitability and accumulation of Na^+^ and Ca^2+^ after ischemic injury by stabilizing the cellular membrane [1, 2]. Recent studies also revealed beneficial effects of K^+^ channel blockade in the ischemic brain [3, 4]. Amiodarone blocks the delayed rectifier potassium current and ATP-sensitive and Ach-sensitive potassium currents, and it prolongs the cellular action potential duration. Because mitochondrial K_ATP_ channels play a crucial role in pharmacological preconditioning effects against ischemic insult [19, 20], blockade of the K^+^ channel can be detrimental. However, amiodarone blocks K_ATP_ channels but has no effect on mitochondrial K_ATP_ channels [21]. Interestingly, some studies have revealed that inhibition of K_ATP_ channels creates a neuroprotective effect through the effect on microglia [4]. Effects of Na^+^-K^+^-ATPase and Na^+^/Ca^2+^ exchanger blockade on the ischemic brain are unclear and controversial [22, 23]. Depending on the cellular electrophysiological condition, these effects can be both harmful and neuroprotective. With a shortage of ATP, inactivation of Na^+^-K^+^-ATPase helps to preserve cellular ATP. If the ATP shortage is critical, however, inactivation of Na^+^-K^+^-ATPase may result in the further accumulation of cellular Na^+^ and Ca^2+^ and exacerbate neuronal death [1]. Na^+^/Ca^2+^ exchanger, an antiporter membrane protein, transports Ca^2+^ out as Na^+^ enters the cell [24]. In ischemic states with accumulated intracellular Na^+^, Na^+^/Ca^2+^ exchanger promotes in the reverse mode, which produces an opposite transporting direction (Ca^2+^ influx and Na + efflux) [25]. The mode in which the Na^+^/Ca^2+^ exchanger operates depends on the Na^+^ and Ca^2+^ transmembrane gradient. These characteristics and the complexity of Na^+^/Ca^2+^ exchanger may explain differing results, indicating both neuroprotective and harmful effects of Na^+^/Ca^2+^ exchanger blockers on ischemic brain injury [22, 23].

Non-electrophysiological effects of amiodarone include inhibitory effects on beta-adrenergic receptors [26]. Blockade of beta-adrenergic receptors has been reported to exert neuroprotective effects possibly by decreasing oxygen consumption, platelet aggregation, and Ca^2+^ influx [27]. In addition to its inhibitory effect on beta-adrenergic receptors, amiodarone also prevents excessive pro-inflammatory cytokine production [28–31] and suppresses reactive oxidative stress [30–32], which can also lead to neuroprotection against ischemic brain injury. Due to its complex pharmacological profile, it is difficult to attribute the neuroprotective effect of amiodarone observed in this study to one simple mechanism. However, the present results suggest that systemically administered amiodarone prior to ischemic brain injury has neuroprotective effects, at least in part, via blocking the sodium channels. We also used isoproterenol to offset the inhibitory effect of amiodarone on beta-adrenergic receptors. However, isoproterenol did not inhibit the neuroprotective effect of amiodarone, indicating that blockade of beta-adrenergic receptors and decrease in oxygen consumption were not likely the mechanisms underlying the neuroprotective effect of amiodarone observed in the present study.

We could not identify a neuroprotective effect of amiodarone post-treatment. It is reported that post-treatment with Na^+^ [2], Ca^2+^ [18], and K^+^ blockers [3, 4], beta-blockers [27], anti-inflammatory drugs [33, 34], and anti-oxidants [35] provides neuroprotection in experimental models of ischemic stroke. One possible reason for this discrepancy is that amiodarone could not reach the ischemic site due to the permanent coagulation of the MCA that did not produce reperfusion. Different results might be obtained by studies using brain ischemia/reperfusion models.

The clinical significance of our study is that systemically administered amiodarone prior to ischemic brain insult has the potential to improve neurological functional outcomes. Amiodarone is one of the most commonly prescribed drugs for life-threatening and refractory arrhythmias, and it is currently a second-line drug for the treatment of ventricular fibrillation during cardiopulmonary resuscitation [36]. Cardiac arrest leads to brain ischemia, and neurological function and recovery after resuscitation are of the greatest concern. We used 50 mg/kg of amiodarone, which is estimated to be equivalent to a cardiac arrest dosage for an adult human (300 mg) based on body surface area [37].

This study had several limitations. Since amiodarone has a complex pharmacological profile, there remains a possibility that other mechanisms are involved in the neuroprotective effect of amiodarone observed in this study. Furthermore, amiodarone could have diverse roles and exert different effects based on various factors or the severity of brain injury. Although there was no statistical difference in blood pressure among the groups, there was a trend toward lower blood pressure in the pre-treatment and post-treatment groups. Hypotension is a major side effect of single-bolus amiodarone [38] that can largely affect brain injury severity. We used relatively young male mice that had no degenerative vascular or cellular changes. Future studies should test these findings in aged mice and female mice with different menopausal states, as sex and menopausal state can significantly affect stroke outcomes [39]. In this study, we conducted infarct volume analysis 48 h after MCAO. However, considering its long elimination half-life and various side effects, the long-term neurological outcomes and effects of amiodarone on ischemic stroke need to be determined.

## Conclusions

Amiodarone pre-treatment, but not post-treatment, attenuates ischemic brain injury and improves functional outcomes without affecting heart rhythm and blood pressure. The neuroprotective properties of amiodarone demonstrated in the present study may be explained by its complex pharmacological profile as a multiple ion channel blocker. The results of the present study may indicate that systemically administered amiodarone prior to ischemic brain insult has the potential to improve neurological outcomes. Further studies are needed to elucidate the underlying mechanisms and therapeutic time windows of amiodarone for ischemic brain injury.

## Abbreviations

ATP: Adenosine triphosphate
ATPase: Adenylpyrophosphatase
MCA: Middle cerebral artery
MCAO: Middle cerebral artery occlusion
TTC: 2,3,5-Triphenyltetrazolium chloride
